# Role of Myeloperoxidase in Patients with Chronic Kidney Disease

**DOI:** 10.1155/2016/1069743

**Published:** 2016-04-03

**Authors:** Bojana Kisic, Dijana Miric, Ilija Dragojevic, Julijana Rasic, Ljiljana Popovic

**Affiliations:** ^1^Institute of Biochemistry, Medical Faculty Pristina, Kosovska Mitrovica 38220, Serbia; ^2^Institute of Pharmacology, Medical Faculty Pristina, Kosovska Mitrovica 38220, Serbia; ^3^Institute of Pathophysiology, Medical Faculty Pristina, Kosovska Mitrovica 38220, Serbia

## Abstract

Chronic kidney disease (CKD) is a worldwide public health problem. Patients with CKD have a number of disorders in the organism, and the presence of oxidative stress and systemic inflammation in these patients is the subject of numerous studies. Chronic inflammation joined with oxidative stress contributes to the development of numerous complications: accelerated atherosclerosis process and cardiovascular disease, emergence of Type 2 diabetes mellitus, development of malnutrition, anaemia, hyperparathyroidism, and so forth, affecting the prognosis and quality of life of patients with CKD. In this review we presented the potential role of the myeloperoxidase enzyme in the production of reactive/chlorinating intermediates and their role in oxidative damage to biomolecules in the body of patients with chronic kidney disease and end-stage renal disease. In addition, we discussed the role of modified lipoprotein particles under the influence of prooxidant MPO intermediates in the development of endothelial changes and cardiovascular complications in renal failure.

## 1. Introduction

Chronic kidney disease (CKD) is a widespread health problem. CKD progresses irreversibly and may lead to end-stage renal disease (ESRD). CKD and ESRD are linked to an increased risk of mortality, cardiovascular complications and comorbidities, and high costs for the treatment of renal failure with dialysis or transplantation. Chronic kidney disease is characterized by the accumulation and/or deficit of various substances in the human body and metabolic disorder caused by the primary disease and therapeutic procedure. Patients with CKD are exposed to increased risk of developing oxidative stress due to metabolic disorders, immune deficiency, and persistent inflammation [1]. Myeloperoxidase (MPO, EC 1.11.1.7) is a heme-containing enzyme found in mammalian neutrophils, where it catalyzes the hydrogen peroxide mediated peroxidation of halide ions and the pseudohalide thiocyanate. Products of these reactions and their secondary metabolites are responsible for killing phagocytized bacteria and viruses [2]. Myeloperoxidase is a 150–165-kDa molecule synthesized during myeloid differentiation that constitutes the major component of the neutrophil azurophilic granules. The enzyme is a homodimer comprising of a pair of light and heavy chains derived from a single gene product with its subunits joined by a single disulfide bridge [3]. Upon phagocyte activation in peripheral blood, tissues, or fluids, MPO is released from leukocytes into both the phagolysosomal compartment and the extracellular milieu [4]. MPO and MPO-derived oxidants may participate as mediators of oxidative modification of biomolecules/tissues and contribute to the development of comorbidities and complications in patient with CKD.

## 2. Oxidative Stress and Inflammation in Chronic Kidney Disease

Previous studies have shown that patients with chronic kidney disease (CKD) are exposed to a number of prooxidants that influence the occurrence of complications and poor outcomes. When increased production and/or inadequate removal of reactive oxygen species (ROS) overwhelm the redox homeostasis of the body, oxidative stress develops. The presence of toxin with prooxidant properties, malnutrition, systemic inflammation, weakened antioxidant defense system, drugs, and side effects of dialysis contribute to the disruption of redox homeostasis and development of oxidative stress in patients with CKD [5]. Reactive oxygen species play an important role in pathogenesis and progression of chronic kidney disease, being responsible for many complications that accompany this pathological condition, such as changes in the vasculature, endothelial dysfunction, atherosclerosis, and cardiovascular disease [6]. Results of numerous studies indicate an increase in concentration of markers of oxidative damage to biomolecules in plasma of patients with CKD. Among the most frequently studied are markers of oxidative damage to proteins (3-nitrotyrosine, carboxymethyllysine, and advanced oxidation protein products), lipids (malondialdehyde, 4-hydroxynonenal, lipohydroperoxides, oxycholesterols, F2-isoprostanes, and oxidized-LDL), and nucleic acids (DNA oxidation and RNA oxidation) [7].

Although dialysis provides a certain quality of life in patients with end-stage renal disease, long-term dialysis therapy causes many complications, probably due to imbalance between generation and elimination of reactive oxygen species. Materials of the hemodialysis membrane may affect both the formation of reactive species by activating polymorphonuclear cells and monocytes and the weakening of antioxidant capacity of the body due to the loss of hydrophilic antioxidants during dialysis, the consumption of liposoluble antioxidants, changes in the lipid composition of biological fluids, and/or deficit of coenzyme and antioxidant enzyme dysfunction [8].

Persistent inflammation is highly prevalent in CKD as a result of numerous disorders [9]. Although the true reasons are not fully elucidated, the immune system disorders in CKD include immunodeficiency and activation of immunocompetent cells, T lymphocytes, and monocytes and the production of proinflammatory cytokines by activated monocytes (TNF-*α*, IL-1*β*, IL-6, etc.) as well as the production of chlorinated oxidants (such as HOCl) by activated neutrophils. In the Chronic Renal Insufficiency Cohort (CRIC) study [10], the inverse correlation between glomerular filtration rate (GFR) and biomarkers of inflammation (IL-1*β*, IL-1 receptor antagonist, IL-6, TNF-*α*, C-reactive protein, and fibrinogen) was demonstrated as well as the direct correlation between kidney function and albuminuria. It was also found that the higher concentrations of acute phase reactants (C-reactive protein, CRP) and proinflammatory cytokines (especially IL-6) strongly predicted cardiovascular morbidity and mortality in patients with CKD [11].

Hemodialysis (HD), as one of the therapeutic procedures performed on patients with renal failure, is believed to contribute to chronic inflammation due to exposure of blood to bioincompatible system causing activation of circulating phagocytes [12]. Hemobioincompatibility depends on the nature of the dialysis membrane and the possible endotoxin contamination of dialysate [8]. Activated neutrophils and monocytes produce reactive intermediate compounds that contribute to oxidative imbalance and development of proinflammatory oxidative stress responses where myeloperoxidase plays an important role. The increase in neutrophil degranulation products, myeloperoxidase and elastase, in HD patients was confirmed as compared to healthy people [13]. Production of oxidants starts upon activation of neutrophils, monocytes, and macrophages by proinflammatory mediators (IL-1*β*, IL-6, and TNF-*α*) when the activity of the NADPH oxidase (NOX-2) enzyme is enhanced. NADPH oxidase catalyzes the one-electron reduction of molecular O_2_ and the formation of superoxide anion radical (O_2_^∙−^), where NADPH serves as electron donor [14]. When phagocytic cell is activated, NADPH oxidase accumulates in vesicles which bind to the cell membrane, and superoxide anion radical (O_2_^∙−^) is released in extracellular space or phagocytic vesicle.

## 3. Myeloperoxidase as a Source of Oxidants in CKD

**Figure 1:**
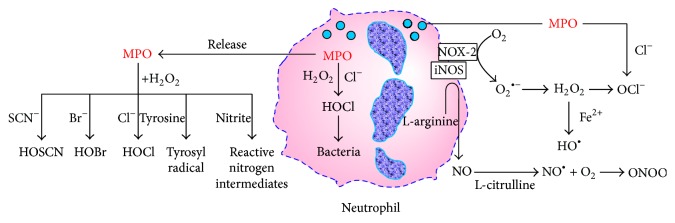
Reactive intermediates species formed by myeloperoxidase (modified from [17]).

Myeloperoxidase is a heme-containing enzyme, involved in oxygen-dependent mechanisms of microbicidal activity of professional phagocytes. MPO is most abundantly present in azurophilic granules of neutrophils, followed by monocytes and some macrophage subpopulations including resident tissue macrophages such as peritoneal macrophages, microglia, and Kupffer cells [15]. MPO catalyzes the reaction of hydrogen peroxide (H_2_O_2_), formed by dismutation of O_2_^∙−^, and halide and pseudohalide ions (Cl^−^, Br^−^, and SCN^−^) to the corresponding hypohalous acids (hypochlorous acid, HOCl; hypobromous acid, HOBr; and hypothiocyanous acid, HOSCN) [16] and formation of other reactive intermediates such as reactive nitrogen species and tyrosyl radicals (Figure 1). Oxidants produced by MPO are microbicidal factors and play an important role in innate immune response defending the body against bacteria, parasites, viruses, and other agents [17]. However, HOCl and other MPO-derived oxidants participate as mediators of oxidative damage to biomolecules, including proteins, nucleic acids, lipids, and carbohydrates, thus damaging the host tissue. In that way they may initiate and contribute to the development of atherosclerosis, endothelial dysfunction, cardiovascular, and other complications in patients with CKD [12]. HOCl can induce dysfunction of endothelial cells and affect endothelial function by decreasing the adhesiveness of extracellular matrix proteins for endothelial cells [18] and by converting matrix metalloproteinases into their active form, thus destabilizing the vascular and tissue environment surrounding endothelial cells [19].

Neutrophils are thought to be the main mediators of vascular inflammation [20]. Given that extracellular MPO is capable of catalyzing peroxidation of lipoproteins and other molecules in the blood, it is considered to be the main driving force for enzymatic lipid peroxidation* in vivo*. Lipid peroxidation of plasma lipoproteins possibly contributes to the development of atherosclerosis, commonly present in CKD patients. Due to the extremely cationic character, MPO can easily bind to the negatively charged structures, that is, bacteria, endothelial cells, cytokeratin-1, polyanionic matrix glycosaminoglycans, apolipoproteins apoA-1 and apoB-100, ceruloplasmin, *α*1-antitrypsin, and albumin [21]. Hypochlorous acid formed through MPO-catalyzed oxidation of chlorine ions is a very strong oxidizing agent; that is, it is a strong oxidant with chlorinating properties [17]. Although HOCl is synthesized predominantly at the site of inflammation within phagocytic vacuole, the surrounding tissues can also suffer the adverse effects when HOCl leaks from incompletely closed phagocytic vacuole.

## 4. MPO-Derived Oxidants and Protein Damage in CKD

Under* in vivo* and* in vitro* conditions, HOCl can react with many biomolecules, with molecules that contain thiols, nitrogen compounds, or unsaturated double carbon bonds. In reaction with protein amino acid residues HOCl forms unstable mono- and dichloramines, which rapidly decompose forming aldehydes. The reaction of the HOCl with the tyrosine residues of proteins gives rise to 3,5-dichlorotyrosine (3,5-dichloro-Tyr) and 3-chlorotyrosine (3-chloro-Tyr) [22]. Glutathione sulphonamide is a specific biomarker of MPO/HOCl production* in vivo*, as a product of HOCl-mediated glutathione oxidation [23]. Reactions of hypohalous acids with proteins contribute to protein unfolding and enzyme inactivation. Products of protein oxidation formed during the activity of phagocytes are considered as specific markers of MPO activity, and their increase in plasma indicates enhanced oxidative stress in CKD patients [13].

Albumin is the most abundant and the most important blood plasma protein. In physiological conditions, serum albumin constitutes about 50–60% of total plasma proteins. Human serum albumin is Mr 65 kDa protein, composed of 585 amino acid residues and one free thiol (Cys 34) [24]. Albumin and low-molecular antioxidants of the blood plasma protect extracellular fluids from the oxidants generated by phagocytes. Also, albumin and plasma proteins, transferrin and ceruloplasmin, which bind transition metals and prevent them from participating as catalysts in reactions that lead to production of reactive species, are the main antioxidants in the extracellular environment. Albumin is a powerful scavenger of oxidants in human plasma that has the ability to inhibit hydroxyl radicals, peroxyl radicals, and HOCl. Also, plasma proteins are very sensitive to the effects of reactive species and permanent exposure of albumin to oxidative stress causes its conformational and functional changes, thus affecting its biological properties. Sulfhydryl group (–SH) of albumin is very sensitive to the influence of HOCl and chloramine. Modified albumin irreversibly losses its biological properties. In patients undergoing maintenance HD the uremic milieu and therapeutic procedure itself, namely, dialysis, contribute to modification of albumin. Structural modifications of serum albumin affect its function in terms of the loss of ligand-binding capacity and other antioxidant properties.

Hypoalbuminemia present in patients with CKD is probably the result of combined effect of inflammation and inadequate protein and calorie intake. Inflammation reduces albumin concentration through cytokine production, reduces protein intake, and increases endogenous albumin catabolism by inducing anorexia. Since the serum albumin is an important antioxidant in the blood and extracellular environment, hypoalbuminemia contributes to the occurrence and development of oxidative stress in patients with CKD. In the human body albumin serves as a reservoir for NO, so that hypoalbuminemia also reduces the bioavailability of NO and the endothelium-dependent vasodilatation. Some studies have shown that hypoalbuminemia in patients undergoing hemodialysis is unfavourable prognostic factor associated with the development of coronary heart disease [25] and also that the level of albumin in plasma has a significant effect on the survival in hemodialysis patients [26].

Advanced oxidation protein products (AOPP) were first proven biochemically and immunochemically in studies of Witko-Sarsat et al. [27] in plasma of hemodialysis patients. From chemical perspective, AOPP consist of various molecular aggregates of oxidatively modified proteins and are present as low-molecular weight AOPP (molecular weight of about 60 kDa) and high molecular weight AOPP (molecular weight of about 600 kDa). The AOPP are dominated by 3-chlorotyrosine, dityrosine, and 3-nitrotyrosine modifications, which confirm the assumption that under* in vivo* conditions AOPP are generated by the action of hypochlorous acid and chloramine produced by MPO. This assumption is confirmed by the evidence that the level of AOPP in plasma correlates with markers of monocyte activation, such as neopterin and TNF-*α* [28]. Biochemical analyses showed that the largest part of the serum AOPP consists of oxidized albumin, and its modification depends on MPO chlorination activity [29]. Polypeptide chain and its side groups are subject to oxidative changes. Also, in patients with CKD, uremic toxins contribute to production of AOPP because they stimulate monocyte-driven inflammatory process [28].

Chronically elevated blood urea, with the accumulation of toxic waste products inherent to a decline in glomerular filtration rate, has been proposed to contribute to the enhanced cardiovascular risk associated with CKD. High levels of urea in CKD facilitate posttranslational modification of proteins through a protein carbamylation [30]. Carbamylation of proteins results from nonenzymatic chemical modification by isocyanic acid derived from urea and an alternative route, the MPO-catalyzed oxidation of thiocyanate. Clinical studies have shown that measurement of circulating levels of protein carbamylation predicts incident cardiovascular risks in patients with CKD and in patients with end-stage renal disease undergoing hemodialysis [31]. Carbamylated albumin appears to be proinflammatory, carbamylated proteins are highly enriched in atherosclerotic plaques, and carbamylated LDL may be a pathogenic ligand for foam cells [32]. Many studies show that the carbamylation of proteins catalyzed by the leukocyte MPO is a dominant mechanism for carbamylation within human atherosclerotic lesions [33]. Urea, which is present in human body as a waste product of protein catabolism, slowly decomposes spontaneously in aqueous solutions forming cyanic acid (and its conjugate base, cyanate). Cyanic acid (HOCN) is in equilibrium with its reactive form, isocyanic acid (HNCO). The plasma concentration of HNCO in healthy individuals is estimated to be ~50 nmol/L but can reach 150 nmol in patients with CKD [31]. The active form of cyanate, isocyanic acid, is capable of interacting with NH_2_ groups of proteins and especially with the *ε*-NH_2_ groups of lysine residues, generating homocitrulline (*ε*-N-C-lysine) [4]. The cyanate may also be generated via enzyme catalyzed oxidation of the pseudohalide thiocyanate (SCN^−^) by MPO [31]. The SCN^−^ is a favoured substrate for MPO, with estimates that up to 50% of the H_2_O_2_ consumed by MPO oxidizes SCN^−^ under physiological conditions. Increasing evidence shows that protein carbamylation results in functional impairment of lipoproteins including high-density (HDL) and low-density lipoproteins (LDL) [30]. Although the mechanisms responsible for the formation of dysfunctional HDL are not completely characterized, there is strong evidence to support a role for MPO-derived chlorinating and nitrating oxidants [34].

## 5. MPO as a Modulator of Immune Response in CKD

Chloramines formed by the action of HOCl are toxic and inactivate the natural antiproteinase, such as *α*1-antitrypsin, while* in vitro* HOCl causes fragmentation of immunoglobulin molecules, suggesting that oxidants generated by MPO can compromise the adaptive host immune response. In addition, activated neutrophils secrete numerous proteases, including cathepsin G. This serine protease plays a role in the release of cytokines, activation of receptors, and degradation of tissue proteins.

There is evidence that MPO also functions as an autocrine modulator of neutrophil function, by recruiting previously unstimulated neutrophils. Extracellular MPO adheres to cell membrane via CD11b/CD18 neutrophil integrins. It is followed by tyrosine phosphorylation and activation of p38 MAP kinase, translocation of nuclear factor *κ*B, and elevated expression of fibronectin and CD11 integrin as well as increased production of superoxide anion radical (O_2_^∙−^) due to activation of membrane NADPH oxidase [35]. This kind of recruitment of new neutrophils increases the production of oxidants in the vascular compartment during inflammation. In addition, MPO can adhere to leukocytes via binding to CD11B/CD18 which may contribute to the proinflammatory effect of MPO through the accumulation of leukocytes at the inflammation site [15]. There are also reports that even enzymatically inactive MPO may affect the activation of endothelial cells and the production of cytokines, IL-6 and IL-8 [36], which is another proinflammatory effect of MPO.

## 6. MPO Genetic Polymorphism and CKD

It is well known that a single-nucleotide G to A polymorphism can occur in the –463 promoter region of MPO gene, located on chromosome 17, associated with altered MPO expression. The presence of G-allele rather than A-allele increases the expression of MPO, which can be the reason of higher enzyme activity in atherosclerosis as well as several malignant, degenerative, and inflammatory diseases. However, the results of clinical studies regarding MPO functional polymorphism and risk factors of kidney damage seem to be controversial. The study of Cayley Jr. et al. [37] has shown that predominantly GG genotype was associated with higher levels of circulating MPO and increased risk of cardiovascular disease. GG genotype was also associated with increased incidence of cardiovascular disease in predialysis CKD patients [38]. Moreover, peripheral neutrophils of GG genotype showed increased production of ROS [39]. Still, the same study reported no differences in MPO genotypes frequencies between healthy controls and CKD patients, with exception in those with hypertensive nephrosclerosis who showed slightly increased frequency of GG genotype [39]. On the other hand, Liu et al. [40] have shown that the presence of A-allele was associated with almost doubled risk of hypertension, which is one of the most important causative factors leading to CKD, while among African American patients with systemic lupus erythematosus the low expression of A-allele was associated with a higher risk of lupus nephritis development [41].

## 7. MPO and Renal Replacement Therapy in CKD

In conservatively treated clinically stable CKD patients, serum MPO was reported to be normal [42] and even decreasing with aggravation of kidney function [43]. In patients who were already on renal replacement therapy, either HD or continuous ambulatory peritoneal dialysis (CAPD), basal serum MPO activity or concentration was reported to increase, with the highest values found in CAPD [28, 44, 45]. Moreover, in patients undergoing maintenance HD treatment the higher basal serum MPO was associated with inflammation, advanced atherosclerosis, and poorer prognosis [44, 46]. On the other hand, basal serum MPO activity in HD patients was in several studies within the range of healthy controls [47, 48], except in cases with arteriovenous fistula thrombosis [49]. These discrepant results probably reflect many confounding factors, such as the duration of HD treatment, use of different dialysis membranes/reuse of a dialyzer, different proportion of underlying causes of CKD, MPO genetic polymorphisms, comorbidities, vitamin, mineral, and antioxidant supplementation, and lipid lowering drugs. Still, MPO-deficiency was recently demonstrated to retard the progression of CKD in 5/6 nephrectomized mice [50], thereby confirming the role of MPO in kidney damage.

The other question is how extracellular MPO is influenced by dialysis procedure itself, and if so, what would be the consequences? Maintenance HD is currently the most preferable renal replacement modality in CKD patients, followed by CAPD. These procedures certainly bring some relief by removing low-molecular weight toxins and oxidation products from the body and maintaining water and ionic balance. Still, the contact of the blood with bioincompatible dialysis membrane or fluid impurity may provoke degranulation of neutrophils and release of MPO, elastase, and other bioactive molecules into vascular compartment. These compounds may further enhance the production of ROS accompanied by formation of more advanced oxidation products and toxic mediators and contribute to proinflammatory and prothrombotic environment, leading to vascular dysfunction [8]. Maintenance HD treatment is usually performed thrice a week, and MPO repetitively released from neutrophils into vascular compartment is highly likely to play an important role in oxidative stress and complications in CKD [28].

Previous studies have shown that even a single HD session would result in severalfold increase in serum MPO activity or concentration [45, 48, 51], but regardless of the magnitude of increment, MPO would usually return to normal well before initiation of the next HD session (Author's unpublished data). Aside from membrane compatibility, there could be at least two other causes for this increment. Using biocompatible membranes and ultrapure dialysis fluid Rutgers and coworkers [52] examined the change in peripheral blood neutrophil MPO activity after 4 hours of HD in 54 CKD patients and 12 healthy volunteers. The results indicated that, unlike in healthy subjects, a single HD treatment was capable of significantly increasing MPO in neutrophils of CKD patients, not only due to MPO genetic polymorphism, but also due to dialysis procedure itself [52]. The other important, although underestimated, cause of intradialytic increase in serum MPO could be endothelium-bound enzyme, released during HD into circulating blood when patients are anticoagulated with heparin, instead of trisodium citrate [53]. That increase rapidly occurred within the first 10–15 minutes, reaching severalfold higher MPO levels than the baseline, decreased during HD session, although still being elevated after completion of procedure [51, 53].

It is however unclear what the consequences of this tidal change of circulating MPO in maintenance HD patients would be. As a strongly cationic protein MPO may reversibly attach to negatively charged groups expressed on endothelial surface glycosaminoglycan-rich receptors and become transiently removed from the circulating blood. Both unfractionated and low-molecular weight heparins compete with MPO for receptors, and bolus injection of these anticoagulants induces the release of MPO into the blood stream [53]. The detachment of MPO from endothelium could be beneficial for patient, since endothelium-bound enzyme may transverse into intima/media where it can act as a prooxidant and proinflammatory agent implicated in atheromatous plaque development. On the other hand, once released into the bloodstream MPO may disseminate to remote tissues thereby accelerating generalized atherosclerosis.

## 8. MPO, Lipid Disorders, and Lipid Peroxidation in CKD

Cardiovascular diseases and complications are the leading cause of morbidity and mortality in patients with CKD, primarily due to atherosclerotic disease [54]. Lipid oxidation/chlorination by the MPO/HOC1 system could be important player in initiation and progression of atherosclerotic changes [55]. The oxidation of low-density lipoproteins (LDL) by HOCl generated in MPO-catalyzed reaction is thought to be a proatherogenic event which precedes the formation of foam cells, a hallmark of atherosclerotic plaque development [56]. Oxidatively modified lipoproteins have reduced functional ability for reverse cholesterol transport, and thus formed lipid peroxides may influence activation, adhesion, infiltration, and differentiation of monocytes as well as the production of MPO and its oxidizing products.

Dyslipidemia, that is, impaired concentration and structure, as well as the metabolism of lipoproteins, is an important factor that increases the risk of coronary heart disease in patients with CKD [57]. The most common blood lipid disorders in CKD are hypertriglyceridemia and increased concentration of atherogenic triglyceride-rich lipoprotein species, such as very low-density lipoproteins (VLDL) and intermediate-density lipoproteins (IDL), while the concentration of high-density lipoproteins (HDL) is decreased [58]. Decreased activity of lipoprotein lipase (LPL) and slow catabolism of triglyceride-rich lipoprotein particles contribute to the development of dyslipidemia in patients with renal insufficiency. LPL is synthesized mainly in adipocytes and myocytes, but, like MPO, when released into the circulation it becomes attached to endothelial cell surface via heparan-sulfate proteoglycans. LPL is implicated mainly in metabolism of chylomicrons and VLDL, while the hepatic lipase mainly metabolizes IDL and LDL. It is believed that endothelial lipase enzyme is responsible for the lipolytic catabolism of HDL in maintenance HD patients [59]. Numerous factors act as inhibitors of lipolytic enzymes in uremic patients: systemic inflammation, oxidative stress, oxidative modification and carbamylation of apolipoproteins, downregulation of gene encoding lipoprotein lipase, and many others [60]. Apolipoprotein C-III is a potent inhibitor, whereas the apolipoprotein C-II is an activator of lipoprotein lipase, and for the normal functioning lipoprotein lipase requires the presence of apoC-II, as a cofactor [61]. Increased permeability of the glomerular membrane leads to the loss of apoC-II, a lipoprotein lipase activator. A decrease of apolipoprotein C-II/C-III ratio due to a disproportionate increase of apolipoprotein C-III in plasma is a possible cause of lipoprotein lipase inactivation in patients with renal insufficiency [62, 63]. Probably a number of mechanisms are responsible for the increase in the concentration of apolipoprotein C-III in the plasma of these patients. Impaired renal function contributes to the above-mentioned, because the kidneys are involved in the elimination of apoC-III from the plasma. The results of some tests [64] pointed out that the mediators of inflammation in the blood of patients with CKD increase apoC-III gene expression and that inflammation with an increased concentration of apoC-III delays VLDL apoB-100 catabolism and its subclasses [63].

The proportion of LDL, which are considered to have a distinct atherogenic potential, was significantly increased in CKD. There were also qualitative changes in LDL particles that involve oxidative modification of LDL and decrease in HDL protection against LDL oxidation. Oxidized-LDL plays an important role in the development and progression of atherosclerotic changes [65]. In addition to inducing endothelial dysfunction, oxidized-LDL particles exhibit proinflammatory activity including chemotactic effects and expression of macrophage colony-stimulating factors, affecting the adhesion of molecules and monocyte activation [66]. MPO and MPO-derived oxidant products contribute to atherogenesis in patients with CKD [67]. The MPO-derived oxidant products induce oxidation of both protein and lipid components of LDL, resulting in the formation of a modified particle, so-called MPO-dependent oxidized-LDL [68]. Oxidative modification of LDL is not limited to the intima of blood vessel but also occurs within the blood plasma. In the state of persistent inflammation when other antioxidants are deprived or at a highly oxidized state, such as in CKD, increased presence of extracellular MPO could be the major initiator of lipoprotein oxidative modifications. In lipoproteins of blood plasma, as a reflection of the slow oxidation, a small amount of autooxidized fatty acids is always present in the form of relatively stable organic hydroperoxides. In the presence of HOCl, these hydroperoxides are converted into peroxyl radicals and singlet oxygen is produced [69]. The study by Delporte et al. [68] confirmed the presence of higher concentrations of MPO-dependent oxidized-LDL (Mox-LDL), as a potential marker of plasma MPO activity in patients undergoing HD treatment.

Beside apoB-100 modifications, MPO-derived oxidants also induce oxidative changes in lipid components of LDL, causing peroxidation of unsaturated fatty acids in phospholipids and fatty acids esterified with glycerol and cholesterol within LDL particles [70]. Lysolecithin is generated in LDL particle as a result of the lipid peroxidation, and the concentration of esterified cholesterol is reduced, a number of lipid-derived oxidation products are formed, including lipid hydroperoxides, and specific products of linoleate and arachidonate oxidation are formed, including 9-hydroxy-10,12-octadecadienoic acid (9-HODE) and F2-isoprostanes [70, 71].

Several experimental and clinical studies confirmed the accumulation of oxidized low-density lipoproteins (oxLDL) in circulation and renal interstitium in CKD [72].* In vitro* studies indicate that oxLDL stimulate mesangial cells to secrete chemoattractant chemokines that attract monocytes [73]. Animal studies have shown that chronic exposure to oxLDL stimulates collagen synthesis and activation of proinflammatory pathways in kidneys [72].

In addition to the transport of excess cholesterol to the liver for further metabolizing, HDL particles play a role as endogenous inhibitors of inflammation, platelet aggregation, and LDL oxidation. A key cardioprotective feature of HDL particles is their participation in reverse cholesterol transport (using apolipoprotein A-I, an integral part of HDL), which removes excess cholesterol from the cells (including macrophages in the artery wall) and transports it to the liver [74]. In CKD, the level of HDL-cholesterol in the blood is lower compared to those with normal renal function, and there are structural modifications and impairment of the HDL particles function. Evidences suggest that MPO is the main enzyme involved in chlorination and nitration of lipoproteins [75]. Analysing chlorinated apoA-I, it has been found that the loss of its activity was the result of oxidation of several methionine residues and chlorination of a single tyrosine residue Tyr192 [76]. Moreover, the reactive lipid peroxidation products such as malondialdehyde (MDA) can modify and cross-link specific residues in apo-AI, causing functional changes in HDL [77] and contributing to atherogenesis due to impaired cholesterol efflux from macrophages [76].

Reverse cholesterol transport is exacerbated in patients with CKD also due to low activity of lecithin cholesterol acyltransferase (LCAT) and paraoxonase. Reduced activity of LCAT enzyme [78] and at the same time increased activity of cholesteryl ester transfer protein [79] may further decrease HDL in patients with impaired renal function. In CKD patients HDL particles have weakened antioxidative and anti-inflammatory properties, possibly due to reduced activity of HDL-associated enzymes, such as paraoxonase [80]. The amount of oxidatively modified HDL particles (oxHDL) is associated with protein-energy wasting in patients undergoing hemodialysis [81]. The loss of serum proteins in dialysis effluent can stimulate the synthesis of albumin and other proteins of the liver, including also a lipoprotein-enriched cholesterol.

## 9. Reactive Nitrogen Species in Chronic Kidney Disease

In the state of inflammation in the body of patients with CKD, oxidative stress is followed by the effects of nitrogen stress. Nitric oxide (NO^∙^) has a significant role in the regulation of vascular tone and arterial tension, prevents platelet aggregation and adhesion of leukocytes to the wall of the blood vessel (by downregulation of endothelial adhesion molecules), prevents the proliferation of smooth-muscle cells of blood vessels, but also exerts adverse effects due to increase in concentration or changes in the oxidation state [82]. NO synthesis is carried out by the influence of the enzymes nitric oxide synthases (NOS), which in the presence of molecular oxygen reduce the substrate L-arginine to L-citrulline. However, the bioavailability of NO in the body depends not only on its synthesis, but also on its degradation mediated by ROS, particularly superoxide anion (O_2_^∙−^) [83]. Nitric oxide rapidly reacts with superoxide radical (O_2_^∙−^) forming the highly reactive peroxynitrite (ONOO^−^), which is a reactive compound of nonradical type and can directly start the process of lipid peroxidation, or indirectly, by reacting with reduced glutathione (GSH), an important factor for antioxidant defense. Peroxynitrite and its protonated form (peroxynitric acid, ONOOH) are strong oxidants; therefore, peroxynitrite leads to nitrosative and oxidative modifications to proteins, nucleic acids, and lipids. The resultant oxidation products such as 3-nitrotyrosine and lipid hydroperoxides accumulate in the fibrotic kidneys as well as in the plasma of patients with chronic kidney disease, suggesting that oxidative stress intensifies during the progression of kidney disease [84]. MPO can catalyze the oxidation of NO and/or its metabolite NO_2_^−^ to the more reactive nitrogen species and thus contribute to a thiol nitrosation and protein tyrosine nitration [85].

Researching endothelial dysfunction in patients undergoing dialysis, which is prior to structural changes in the vascular tree and the consequent clinical changes in blood vessels, Stenvinkel et al. [86] confirmed that endothelial dysfunction is associated with reduced NO bioavailability and increased concentration of its endogenous inhibitor: asymmetric dimethylarginine (ADMA) concentration in serum. Namely, ADMA is synthesized when L-arginine incorporated into proteins is methylated by the enzyme protein-arginine methyltransferase during posttranslational protein modification [87]. When methylated proteins are hydroxylated during transport of proteins, ADMA residues are released. Due to the very similar structure to L-arginine, ADMA may act as an endogenous competitive inhibitor of NO synthase [88]. In patients undergoing hemodialysis, elevated level of ADMA is associated with a higher risk of cardiovascular disease and mortality [89]. Also, the increased concentration of ADMA is associated with increased morbidity, mortality, and graft failure in kidney transplant recipients [90].

Along with the formation of ADMA, its inactive stereoisomer symmetrical dimethylarginine (SDMA) is created. SDMA does not directly inhibit NO synthase but is a competitor of L-arginine transport and impairs L-arginine uptake from the loop of Henle, which limits the availability of L-arginine to NO synthase [91]. During the study by Schepers et al. [92], it was proven that SDMA has a proinflammatory effect through stimulation of generating ROS in monocytes, which can be a driver of vascular damage.

## 10. Conclusion

Examination of the effect of oxidative stress on the occurrence and development of CKD is important for assessing the degree of damage, possibility of slowing further development of the disease, and delaying the development of complications and comorbidities. Activation of neutrophils, release of myeloperoxidase, and the consequent formation of reactive/chlorinating intermediate products contribute to persistent inflammation and oxidative damage to biomolecules in the body of patients with CKD. MPO-derived reactive species (e.g., hypochlorous acid) may influence cell function and contribute to tissue damage. Oxidatively modified lipids and proteins contribute to the initiation and propagation of atherosclerotic changes and the development of cardiovascular complications in CKD. Despite progress made in understanding the complex mechanisms of MPO and MPO-derived reactive intermediates activities, further research in order to determine the exact role in the development of oxidative stress and inflammation in renal failure would be certainly justified.
